# The Thomsen-Friedenreich Antigen: A Highly Sensitive and Specific Predictor of Microsatellite Instability in Gastric Cancer

**DOI:** 10.3390/jcm7090256

**Published:** 2018-09-05

**Authors:** Stefan Mereiter, Karol Polom, Coralie Williams, Antonio Polonia, Mariana Guergova-Kuras, Niclas G. Karlsson, Franco Roviello, Ana Magalhães, Celso A. Reis

**Affiliations:** 1 I3S—Instituto de Investigação e Inovação em Saúde, University of Porto, 4200-465 Porto, Portugal; amagalhaes@ipatimup.pt; 2 IPATIMUP—Institute of Molecular Pathology and Immunology, University of Porto, 4200-135 Porto, Portugal; 3 General Surgery and Surgical Oncology Department, University of Siena, 53100 Siena, Italy; surgoncolclub@gmail.com (K.P.); franco.roviello@gmail.com (F.R.); 4 Department of Surgical Oncology, Medical University of Gdansk, 80-211 Gdańsk, Poland; 5 Ariana Pharmaceuticals, 75016 Paris, France; c.williams@arianapharma.com (C.W.); mariana.kuras@free.fr (M.G.-K.); 6 Department of Pathology, IPATIMUP Diagnostics, University of Porto, 4200-135 Porto, Portugal; apolonia@ipatimup.pt; 7 Medical Biochemistry, Institute of Biomedicine, Sahlgrenska Academy, University of Gothenburg, 40530 Gothenburg, Sweden; niclas.karlsson@medkem.gu.se; 8 Faculty of Medicine, University of Porto, 4200-319 Porto, Portugal; 9 Instituto de Ciências Biomédicas Abel Salazar, University of Porto, 4050-313 Porto, Portugal

**Keywords:** glycosylation, mucin type glycans, gastric cancer, microsatellite instability, cancer biomarker, *O*-glycan truncation

## Abstract

Microsatellite instability (MSI) is a distinct molecular subtype of gastric cancer. In recent years, the clinical consequences of MSI and the therapeutic opportunities to target this peculiar cancer subtype became evident. However, despite the importance of MSI for the stratification of patients, the time and resources required for diagnosis still present an obstacle. In an attempt to identify a new marker for MSI in gastric cancer, we evaluated the expression of five cancer-associated glycan epitopes in a cohort of 13 MSI and 17 microsatellite stable (MSS) cases. Our analysis revealed a highly significant (*p* < 0.001) association between the expression of the Thomsen-Friedenreich (TF) antigen and MSI status. Hence, we present here the identification of the first single marker for MSI in gastric cancer, excelling with a specificity of 94% (16/17), sensitivity of 69.2% (9/13), negative predictive value of 80% (16/20), and positive predictive value of 90% (9/10). The TF antigen, detected by simple antibody-based assays, is highly specific for carcinoma being undetectable in gastric healthy and premalignant epithelia. This finding lays the basis for new studies and holds promise in improving the rapid identification of MSI in the clinical setting.

## 1. Introduction

Gastric cancer is a heterogeneous disease that requires multidisciplinary treatment [1]. Currently, new molecular classifications for gastric cancer have been proposed by TCGA (The Cancer Genome Atlas) and ACRG (Asian Cancer Research Group) in which microsatellite instability (MSI) is described as a distinct subgroup [1,2]. According to TCGA, the MSI subgroup is linked with hypermutation, gastric CpG island methylator phenotype, MLH1 silencing, and mitotic pathways [1]. In the last few years, many publications described MSI from a clinical point of view, being found in 5.6% to 33.3% of all gastric cancers and strongly associated with female sex, older age, intestinal histotype, middle/lower gastric position, N0 status, and TNM stage I/II with favorable overall survival [1,2,3,4,5]. It is important to underline that MSI is not a homogenous group and it has been shown that MSI is a prognostic factor because of the association with tumor localization and Lauren classification [6]. Additionally, it has been found that MSI is linked with PD-L1 expression and several specific drugs are currently used for clinical management of gastric cancer targeting the PD1/PD-L1 immune checkpoint pathway [7]. The molecular diagnosis of MSI gastric cancer is of highest importance, especially before multidisciplinary treatment. It presents different responses to neoadjuvant chemotherapy and requires specific surgical treatment in synchronous metastases, includes the possibility of tailored lymphadenectomy, and stratifies for the application of targeted therapies [3,8].

The transformation of gastric epithelial cells is accompanied by several changes in the protein glycosylation machinery resulting in the aberration of cellular glycosylation, such as the truncation of *O*-linked glycans and the expression of sialofucosylated glycan epitopes [9]. The truncation of *O*-linked glycans has been widely described in various gastrointestinal carcinomas and results in the *de novo* expression of short *O*-glycans such as the Thomsen-Friedenreich (TF or T, Galβ1-3GalNAcα1) antigen, Thomsen-nouvelle (Tn, GalNAcα1) and its sialylated form sialyl Tn (STn, Neu5Acα2–3GalNAcα1) [9]. In addition, the expression of complex sialofucosylated structures, such as sialyl-Lewis A (SLe^a^, Neu5Acα2–3Galβ1–3 [Fucα1–4] GlcNAcβ-) and sialyl-Lewis X (SLe^x^, Neu5Acα2–3Galβ1–4 [Fucα1–3] GlcNAcβ-) has been frequently described in gastric tumors with an important role in cancer progression and metastasis formation [9].

The TF antigen, also known as core 1 structure, is an intermediate structure during the maturation of mucin-type *O*-glycans within the Golgi apparatus. Under physiologic conditions, the TF antigen is below the limit of detection because it is modified with additional saccharides and made inaccessible due to surrounding larger glycans [10,11]. It is only found in the luminal surfaces of the pancreatic duct, kidney distal tubule and kidney collecting duct [11]. In addition, macrophages of the thymus, spleen and lymph nodes carry the TF antigen, suggesting a potential immunologic role [11]. Importantly, the TF antigen is neither found in the healthy gastric epithelium nor in gastric pre-malignant conditions [12]. In gastric cancer the TF antigen is expressed in considerable amounts in around 21% of the tumours, associating with higher immune response [12].

As molecules that are secreted into circulation and due to their expression specificity for malignant cells, aberrant glycans and glycoconjugates have a long-lasting history as cancer biomarkers [13]. We report here the analysis of TF, Tn, STn, SLe^a^ and SLe^x^ expression in gastric carcinomas in a cohort of 30 patients, 13 with MSI high and 17 with MSI low or negative status, revealing a novel strong association between TF expression and MSI status. Our data suggest the TF antigen as a single specific and sensitive marker for the MSI status.

## 2. Material and Methods

### 2.1. Immunohistochemistry Profiling of Glycan Markers

Formalin-fixed paraffin embedded (FFPE) tissue samples from 30 gastric carcinomas were provided by the department of surgical oncology of the University of Siena (Italy). All procedures were performed after patients’ written informed consent and approved by the local ethical committee. FFPE blocks were cut into 3 μm sections and mounted onto glass slides. The sections were dewaxed, rehydrated and endogenous peroxidases were inactivated with 3% hydrogen peroxide (H_2_O_2_) in methanol. Tissue sections were blocked for 30 min with normal rabbit serum in PBS with 10% BSA. The primary antibodies 3C9, specific for the TF antigen [10], B72.3, specific for STn [14], CA19.9, specific for SLe^a^ [15] or CSLEX-1, specific for SLe^x^ [16], were incubated overnight at 4 °C. Biotin-labeled secondary antibodies (Dako, Glostrup, Denmark) were applied for 30 min and the ABC kit (Vector Labs’, Burlingame, CA, USA) for an additional 30 min. Finally, sections were stained by 3,3′-iaminobenzidine tetrahydrochloride (DAB) (Sigma Aldrich, St. Louis, MO, USA) and counterstained with Gill’s hematoxylin solution. Slides were examined using a Zeiss Optical Microscope and the proportion of positive cancer cells within the tumor were estimated by pathologists. The Tn expression data were acquired as we previously reported [17]. For the classification into positive and negative groups, a cut-off of 25% cancer cell positivity was defined for Tn, STn, SLe^a^, SLe^x^ and, due to its higher tumor specificity, 5% for TF.

### 2.2. Microsatellite Instability Analysis

All gastric carcinomas were evaluated for MSI status and detailed clinicopathologic features as previously reported [18]. In brief, all cases were classified according to the AJCC/UICC TNM classification (7th edition). All tumors were additionally classified according to Lauren, WHO and Borrmann. For the evaluation of MSI status, frozen tumors and normal mucosa were used from each patient. After histopathological confirmation, tumoral and constitutional DNA were extracted following the manufacturer’s instructions (Puregene, Gentra Systems, Minneapolis, MN, USA). Mononucleotide repeats were evaluated with fluorescently labelled primer for BAT-26, NR-21, BAT-25, NR-27 and NR-24 (ABI PRISM Primer Pairs; Applied Biosystems, Foster City, CA, USA). These repeats were co-amplified on tumoral and matched constitutional DNA of each patient in a pentaplex PCR using the manufacturer’s protocol (Multiplex PCR, Qiagen, Studio City, CA, USA). The allelic profiles were detected by the automated DNA sequencer ABI PRISM 3100 Genetic Analyzer (Applied Biosystems, Foster City, CA, USA) and the MSI status was determined in accordance to the guidelines of the National Cancer Institute on MSI for cancer detection and familial predisposition. Hence, whenever 2 or more markers showed instability on 5 loci the tumor was considered MSI high. In contrast, when one or no locus was involved MSI low or microsatellite stable (MSS) was diagnosed, respectively. Since clinicopathological characteristics and survival rates of MSI low and MSS are known to be similar [5,18] the two groups were joint to MSS.

### 2.3. Correlation Statistical Analysis of Glycan Markers and MSI Status

The statistical analysis of glycan markers, MSI status and patients clinicopathological features were performed in R statistical software (R Core Team, Vienna, Austria). Fisher’s exact test was applied to assess if there was a significant association between glycan markers and MSI status. Similarly, associations between TF marker status (positive or negative) and clinicopathological categorical descriptors were calculated with fisher’s exact test and chi-squared test for descriptors with more than two groups. Statistically significant discrepancy in medians of demographic and clinical descriptors (i.e., age and survival in months since date of diagnosis) between TF positive and negative groups was analyzed using Mann-Whitney-Wilcoxon test. *p*-values ≤ 0.05 were considered to be statistically significant.

## 3. Results

We analyzed 30 gastric carcinoma cases (13 MSI high and 17 MSS/MSI low) for the expression of TF, STn, SLe^x^ and SLe^a^. Additionally, we have previously evaluated this cohort for the expression of Tn [17]. The expression of each antigen was used for association analyses with MSI status (Table 1), which revealed a highly significant association between the expression of TF and MSI status (*p* < 0.001; Fisher’s exact test). Among the 10 TF positive cases, 9 had MSI, which suggests an unprecedented specificity of the TF antigen for this gastric cancer molecular subtype (Table 1). Our results indicate that the TF antigen has also very good sensitivity, as 16 of the 20 TF negative cases were MSS or MSI low (Table 1). This results in sensitivity and specificity values of 69.2% (9/13) and 94.1% (16/17), respectively. Positive and negative predictive values were 90% (9/10) and 80% (16/20), respectively.

**Figure 1:**
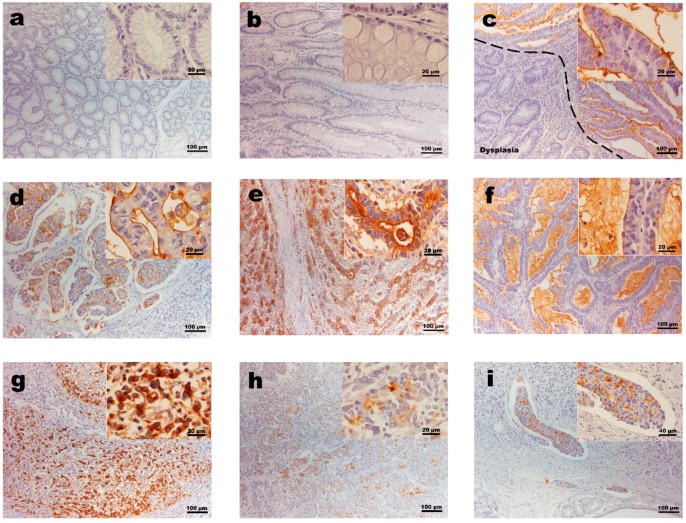
Thomsen-Friedenreich antigen (TF) expression in human gastric tissue samples. (**a**) Gastric mucosa is negative for TF, here represented by histologically normal mucosa adjacent to MSI positive and TF positive tumor. (**b**) Representative image of intestinal metaplasia showing that even goblet cells, which are commonly enriched in truncated O-glycan carriers, were negative for TF. (**c**) TF negative dysplasia next to TF positive carcinoma, showing the specificity of TF for malignant cells. (**d**–**f**) Well-differentiated gastric carcinomas show a typical membranous TF staining at the apical surface, including mucinous secretions. (**g**–**i**) Poorly differentiated gastric carcinomas were dominated by cytoplasmic staining. (**i**) TF positive carcinoma cells within an infiltrated vessel.

The TF epitope was expressed in 10 of the 30 evaluated gastric carcinoma cases. The mucosas adjacent to tumors, including normal glands and highly inflamed regions, as well as intestinal metaplasia and dysplasia, were completely negative, underlining the absence of this glycan marker from non-malignant and pre-malignant conditions (Figure 1a–c).

Among the positive cases, the staining was typically membranous and found on average in around 30% of all cancer cells of the tumor. In well-differentiated gastric carcinomas, the staining was typically at the apical membrane and included secretion (Figure 1d–f). The subcellular localization among poorly differentiated gastric carcinomas shifted typically to cytoplasmic (Figure 1g–j).

We did not find any further statistical significant association between the TF expression and other clinicopathological features (Table 2).

However, the expression of TF seems to associate with good prognosis for the patients, reflected by the increased median patient survival (88 months vs. 31.5 months) and the high proportion of patients being alive 5 years after diagnosis (70% vs. 30%) (Table 2). This improved prognosis is in accordance with expectations, as an on average better survival is known to occur for MSI patients.

## 4. Discussion

Here we present for the first time strong evidence that the cellular expression of the TF antigen is associated with the MSI gastric cancer subtype. Due to the high amount of glycoconjugates expressed by cancer cells and secreted into circulation, cancer-specific glycan epitopes represent promising targets for biomarker application in blood tests and liquid biopsies. Indeed, most of the established cancer biomarkers currently applied in the clinical setting detect glycan moieties or glycoconjugates [13]. In contrast, current MSI analysis is a resource intensive procedure evaluating either the tumor PCR profile of 5 MSI markers and comparing them to the profile of matching normal DNA or evaluating the absence of at least one of four nuclear expressed markers in tumor sections.

Mismatch repair deficiency (dMMR) testing can be performed by immunohistochemistry in paraffin sections using commercially available antibodies evaluating the expression of four MMR proteins (MLH1, PMS2, MSH2 and MSH6) in tumor cell nuclei or by PCR, as mention above. Usually, MMR status is tested in colorectal cancer specimens for the identification of patients at elevated risk for Lynch syndrome as well as for prognostic stratification. However, recent data has emerged showing that dMMR can have predictive value for immune checkpoint inhibitor therapy, regardless of the cancers’ tissue of origin [19]. In fact, it was approved in the last year by the Food and Drug Administration that pembrolizumab (anti-programmed cell death protein-1 (PD-1)) could be used in any solid tumor with MSI or dMMR that have progressed following prior treatment.

Our data indicate that the TF antigen, as a single marker, is a highly reliable predictor of MSI status in gastric cancer. The application of TF has, therefore, strong potential for the stratification of patients with MSI in a time and cost-efficient manner.

Thus, the TF antigen holds promise to be the long sought-after single biomarker for MSI status in gastric cancer. Moreover, TF has several additional assets which could allow it to be used as serologic biomarker application. Firstly, it is hardly expressed in human tissues, neither under healthy nor under pathologic conditions [11,20]. Secondly, the basal levels of exposed TF are very low in the bloodstream due to naturally circulating anti-TF IgM and IgG antibodies and the rapid clearance of terminally galactosylated glycoconjugates by the liver [21]. Lastly, as a mucin-type *O*-glycosylation it may be carried by a wide range of secreted glycoproteins that are overexpressed in gastric cancer such as MUC1 or CD44. Future studies testing the applicability of TF detecting ELISA based serological assays for the diagnosis of MSI subtype have potential utility in the clinical setting. In addition, other strategies such as the detection of elevated levels of autoantibodies against TF, as it has been frequently described in cancer [21], could have indicative properties for the MSI status.

The TF antigen is a simple glycan antigen that is aberrantly expressed in cancer cells due to alterations in the *O*-glycan biosynthesis pathway. However, the underlying molecular mechanism that leads to the expression of the TF-antigen in this molecular subtype and the phenotypic consequences remain elusive. The expression of TF might facilitate the tumor immune response evasion, since the TF epitope is a potent ligand for galectin-1 and -3, which are known to modulate the immune response [22].

Another possible explanation is that the TF epitope is a byproduct of increased tandem repetitions within the MSI cancer cell genome. The abrogation of an efficient DNA mismatch repair system leads to the increase and creation of new simple sequence repeats, known as microsatellites. The detection of these sequence repeats by PCR assays are the defining feature of MSI tumors. Besides the acquisition of new microsatellites, it has been shown that tandem repeats within variable number of tandem repetition (VNTR) regions are increased in gastric cancer [23]. VNTRs harbor the mucin-type *O*-glycosylation sites in mucin proteins and the sole increase of these tandem repeats has been demonstrated to result in the *de novo* expression of the TF antigen [24]. The fact that in average only 30% of the cancer cells were positive supports the hypothesis that the TF expression does not convey a significant growth advantage to cancer cells but arises as a passenger alteration in cells with defective mismatch repair.

In summary, we presented here for the first time strong evidence that TF is a novel, single marker of MSI in gastric cancer. This finding lays the basis for a new line of research and shows great promise to significantly improve the identification of this distinct cancer subtype in the clinical setting.

## Figures and Tables

**Table 1:** Association Analysis of Aberrant Glycan Epitopes with MSI Status.

Patient	MSI	TF	Tn	STn	SLe^x^	SLe^a^		Fisher’s Exact Test			
1	−	+	+	+	−	−			TF		
2	−	−	+	+	+	+		***p* = 0.00041**	+	−	Σ
3	−	−	+	−	−	+	MSI	+	9	4	13
4	−	−	+	−	−	+		−	1	16	17
5	−	−	+	+	−	−		Σ	10	20	30
6	−	−	+	−	+	−					
7	−	−	+	−	−	+			Tn		
8	−	−	−	−	−	+		***p* > 0.99**	+	−	Σ
9	−	−	+	+	−	+	MSI	+	11	2	13
10	−	−	+	−	−	+		−	13	2	15
11	−	−	+	+	/	+		Σ	24	4	28
12	−	−	/	−	−	−					
13	−	−	+	−	−	+			STn		
14	−	−	+	+	−	−		***p* = 0.713**	+	−	Σ
15	−	−	/	−	−	+	MSI	+	7	6	13
16	−	−	+	+	−	+		−	7	10	17
17	−	−	−	−	−	+		Σ	14	16	30
18	+	+	+	−	−	−					
19	+	+	+	+	+	+			SLe^x^		
20	+	+	+	−	−	−		***p* > 0.99**	+	−	Σ
21	+	+	+	+	−	−	MSI	+	2	11	13
22	+	+	+	−	−	−		−	2	14	16
23	+	+	+	+	−	+		Σ	4	25	29
24	+	+	+	−	−	−					
25	+	+	+	+	−	−			SLe^a^		
26	+	+	+	+	−	+		***p* = 0.063**	+	−	Σ
27	+	−	+	+	−	−	MSI	+	4	9	13
28	+	−	−	−	+	+		−	12	5	17
29	+	−	+	+	−	−		Σ	16	14	30
30	+	−	−	−	−	−					

Table 1 Legend: The expression of five marker of aberrant glycosylation, namely TF, Tn, STn, SLe^x^ and SLe^a^, were evaluated in 13 microsatellite instability (MSI) and 17 microsatellite stable (MSS) gastric carcinomas. Statistical analysis of markers with MSI was performed using Fisher’s exact test, revealing a highly significant association of TF with MSI. Missing values are indicated by (/).

**Table 2:** Clinicopathological Analysis of Gastric Cancer Patients According to TF Status.

Variable	Categories	Total		TF Positive		TF Negative		*p*-Value
		*n*	%	*n*	%	*n*	%	
		30	100%	10	33%	20	67%	
Age	Median value	72.5		76		71.5		0.29
Survival in months	Median value	44.5		88		31.5		0.15
Gender	F	13	43%	5	50%	8	40%	0.70
	M	17	57%	5	50%	12	60%	
MSI status	High	13	43%	9	90%	4	20%	0.0004
	Stable	17	57%	1	10%	16	80%	
Adjuvant Therapy	No	17	57%	8	80%	9	45%	0.12
	Yes	13	43%	2	20%	11	55%	
Survival	*missing value*	*2*	*7%*	0	0%	*2*	*10%*	0.18
	High (≥5 years)	13	43%	7	70%	6	30%	
	Medium (≥2 years & <5 years)	5	17%	1	10%	4	20%	
	Low (<2 years)	10	33%	2	20%	8	40%	
GC Family History	False	17	57%	7	70%	10	50%	0.44
	True	13	43%	3	30%	10	50%	
Primary Tumor	T1	0	0%	0	0%	0	0%	0.58
	T2	7	23%	3	30%	4	20%	
	T3	10	33%	4	40%	6	30%	
	T4	13	43%	3	30%	10	50%	
Regional Lymph Nodes	N0	12	40%	5	50%	7	35%	0.80
	N1	5	17%	2	20%	3	15%	
	N2	7	23%	2	20%	5	25%	
	N3a	2	7%	0	0%	2	10%	
	N3b	4	13%	1	10%	3	15%	
Metastasis	M0	27	90%	9	90%	18	90%	0.99
	M1	3	10%	1	10%	2	10%	
Staging	I	4	13%	2	20%	2	10%	0.82
	II	11	37%	4	40%	7	35%	
	III	12	40%	3	30%	9	45%	
	IV	3	10%	1	10%	2	10%	
WHO	*missing value*	*1*	*3%*	*1*	*10%*	*0*	*0%*	0.37
	Papillary adenocarcinoma	1	3%	0	0%	1	5%	
	Poorly cohesive	11	37%	3	30%	8	40%	
	Signet-ring cell & mucinous	7	23%	1	10%	6	30%	
	Tubular adenocarcinma	10	33%	5	50%	5	25%	
Borrman Classification	I	2	7%	0	0%	2	10%	0.64
	II	7	23%	2	20%	5	25%	
	III	17	57%	7	70%	10	50%	
	IV	4	13%	1	10%	3	15%	
Lauren Classification	*missing value*	*2*	*7%*	*1*	*10%*	1	5%	0.10
	Diffuse	5	17%	0	0%	5	25%	
	Intestinal	22	73%	8	80%	14	70%	
	Mixed	1	3%	1	10%	0	0%	
Tumor Site	Diffuse	1	3%	0	0%	1	5%	0.73
	Cardia	1	3%	0	0%	1	5%	
	Corpus	10	33%	3	30%	7	35%	
	Antrum	18	60%	7	70%	11	55%	
Vessel infiltration	*missing value*	*3*	*10%*	*0*	*0%*	*3*	*15%*	0.23
	FALSE	14	47%	7	70%	7	35%	
	TRUE	13	43%	3	30%	10	50%	
Nerve infiltration	*missing value*	*6*	*20%*	*3*	*30%*	*3*	*15%*	0.99
	FALSE	16	53%	5	50%	11	55%	
	TRUE	8	27%	2	20%	6	30%	

Table 2 Legend: Description of total cohort (30 patients), TF positive patients (10) and TF negative patients (20), along with comparison of the TF status (negative/positive) with clinicopathological features. Associations between clinicopathologic groups and the two TF marker status groups was calculated using Fisher’s exact test for features with two categories and *χ*² test for features with more than three categories. Difference in medians of age and survival since diagnosis (in months) of the TF status groups was statistically evaluated using Mann-Whitney-Wilcoxon test.

## Abbreviations

dMMR	mismatch repair deficiency
FFPE	formalin fixed paraffin embedded
MMR	mismatch repair
MSI	microsatellite instability
MSS	microsatellite stable
SLe^a^	sialyl Lewis A
SLe^x^	sialyl Lewis X
STn	sialyl Thomsen-nouvelle
TF	Thomsen-Friedenreich
Tn	Thomsen-nouvelle
VNTR	variable number of tandem repetition
